# QTL mapping with different genetic systems for nine non-essential amino acids of cottonseeds

**DOI:** 10.1007/s00438-017-1303-7

**Published:** 2017-03-18

**Authors:** Haiying Liu, Alfred Quampah, Jinhong Chen, Jinrong Li, Zhuangrong Huang, Qiuling He, Chunhai Shi, Shuijin Zhu

**Affiliations:** 1 Department of Agronomy, College of Agriculture and Biotechnology, Zhejiang University, Hangzhou, 310058 People’s Republic of China; 2 School of Agriculture, Yunnan University, Kunming, 650000 People’s Republic of China

**Keywords:** Cottonseed, Amino-acid content, QTLs, Genetic effects, QTL mapping population

## Abstract

Amino acid is an important nutrient resource for both human and animals. Using a set of 188 RILs population derived from an elite hybrid cross of upland cotton cultivars ‘HS46’ × ‘MARCABUCAG8US-1-88’ and their immortal F_2_ (IF_2_) with reciprocal backcrosses BC_1_F_1_ and BC_2_F_1_ (BC) populations in two environments, the QTLs located on the embryo genome and maternal plant genome for nine amino acids of cottonseed were studied across environments. The QTL Network-CL-2.0-seed software was used to analyze the QTLs and their genetic effects for nine amino acids. A total of 56 QTLs for nine amino acids were detected in both populations, with many having over 5% of phenotypic variation. Ten of the total QTLs could be simultaneously found in the IF_2_ and BC populations. For most QTLs, the genetic effects from embryo genome were more important than those from maternal plant genome for the performance of nine amino acids. Significant embryo additive main effects and maternal additive main effect with their environment interaction effects from many QTLs were also found in present experiment. Some QTLs with larger phenotypic variation were important for improving the amino-acid contents in cottonseeds.

## Introduction

Cottonseed is an important by-product of cotton, with about 35% of high-quality protein and 30% of edible oil. In the past, cottonseeds were mainly used as the animal feed. With the decreasing or removing of gossypol in the cottonseeds, the cottonseed protein which consists of all kinds of amino acids could become a potentially important resource for humans or animals (Ji et al. 1985; Huang et al. 2011). With the increasing demand for superior food nutrient quality, crop quality traits such as protein, oil, fatty acid, and amino acid are attracting increasing attention from researchers. The contents of amino acids determine the nutritional quality of crop plants used in the diet of humans and animals.

Quality traits in cottonseeds such as the contents of protein, oil, and amino acids are quantitative in nature. They are mainly under polygenetic control and also influenced by environmental factors. Song and Zhang (2007) found eight major QTLs which controlled the performance of seven amino acids in cottonseed using a BC_1_S_1_ population derived from an elite cross between TM-1 and Hai7124, an interspecific hybridization. Yu et al. (2012) identified quantitative trait loci (QTLs) for cottonseed oil, protein, and gossypol contents using an interspecific hybrid backcross inbred line population, *G. hirsutum* and *G. barbadense*, in which 17 QTLs on 12 chromosomes related to oil content, 22 QTLs on 12 chromosomes to protein content, and three QTLs on two chromosomes to gossypol content were detected. However, these studies were carried out based on one genetic system, the embryo genome only. According to the previous reports, cottonseed traits could be simultaneously controlled by seed nuclear genes, cytoplasmic genes, and maternal nuclear genes (Zhu and Weir 1994; Ye et al. 2003). The results from QTL analysis performed by Liu et al. (2012, 2013) for oil, fatty acid, protein, and gossypol contents showed that the performance of these quality traits was governed by the genetic effects of QTL from both embryo and maternal nuclear genomes at the same time. So far, there are just a few reports on the QTL mapping of amino-acid contents based on the different genetic systems. Liu et al. (2013) reported QTLs for eight amino acids which cannot be synthesized by humans and analyzed their genetic effects from the embryo and maternal nuclear genomes. There are other kinds of amino acids which are important as well for the growth and development of plants, although they can be synthesized by human beings and animals. However, they have not yet been reported.

The genetic main effects including the embryo additive main effect, embryo dominance main effect, and maternal additive main effect of QTLs, as well as their QTL× environment interaction effects from different genetic systems were analyzed in this paper, for nine amino acids including Aspartic acid (Asp), Serine (Ser), Alanine (Ala), Glutamic acid (Glu), Glycine (Gly), Tyrosine (Tyr), Histidine (His), Arginine (Arg), and Proline (Pro). This result might comprehensively reveal the QTLs and their molecular genetic mechanism for these amino acids, which might be help to clone some useful candidate genes related to amino-acid content according to the QTL mapping gene identification and serve in MAS for the improvement of these amino acids in cottonseeds.

## Materials and methods

### Plant materials and field experiment

A set of 188 RILs population derived from a cross of two upland cotton parents, HS46 (P_1_) and MARCABUCAG8US-1-88 (MAR, P_2_), was used to construct research populations, an immortal F_2_ (IF_2_ population) and two reciprocal backcrossing populations (BC populations), in this study. HS46 is a commercial upland cotton cultivar with higher yield and good fiber qualities, and MARCABUCAG8US-1-88 is an upland cotton germplasm with good resistance to multiple adversity. These two parents have wide genetic difference in different traits including amino acids. A whole set of 188 RILs population were kindly supplied by USDA-ARS, Starkville, Mississippi, USA in 1999. It was constructed after successive selfing for eight generations from the intra-specific hybrid between HS46 and MARCABUCAG8US-1-88 through the modified single-hill (bulked progeny row) method. The IF_2_ population which includes 376 individuals was obtained from an incomplete diallel cross among these RILs. The two reciprocal backcross populations containing 376 BCF_1_ were obtained by crossed 188 RILs with P_1_ and P_2_, respectively.

All materials were grown using randomized complete block design with two replications at Sanya, Hainan province of China in 2009 and 2010. There was one row for each plot with 7.0 m length and 0.8 m width.

### Determination of the amino-acid contents

The contents in cottonseeds were determined by NIRS (near-infrared reflectance spectroscopy) analysis. The crossed bolls of IF_2_ population and two BC populations, as well as their parents were manually harvested, then delinted, and dried. Two hundred seeds from each sample were removed from their kernels and ground into powder with the Universal High-speed Grinder DFT-50 (Linda Machinery Company Ltd, Wenlin, Zhejiang Province, China). The powdered samples were dried to equilibrium at 25 °C with a moisture content of about 7% (Huang et al. 2011). About 3 g of cottonseed powder was scanned using a 36 mm inner-diameter ring cup which is attached to the FOSS NIR Systems 5000 (Silver Spring, MD, USA). Each sample was scanned for four times at the range from 1100 to 2498 nm, to obtain the spectrum data. Based on the NIRS Calibration of nine acids in cottonseed (Huang et al. 2011), the spectrum data were used to determine these amino-acid contents with the WINISI 1.04 version software (Infrasoft international inc., Port Matilda, PA, USA).

### Statistical analysis for phenotypic evaluation

Statistical analysis was carried out using SPSS 13.0 software (SPSS for windows, SPSS Inc. Chicago, USA). The samples for each trait were extracted in two replicates for each environment. According to the following formulae (1, 2), values of each environment were averaged and represented as means with standard deviations (SD):

1 $$\bar{X}=\frac{\sum\nolimits_{i=1}^{n}{{{X}_{i}}}}{n}$$

2 $$S=\sqrt{\frac{\sum\nolimits_{i=1}^{n}{({{x}_{i}}-\bar{x}})_{{}}^{2}}{n-1}}$$

where $\bar{X}$ or $\bar{x}$ is the mean of each sample; ${{X}_{i}}$ is the observed value of an individual; *n* is the number of samples measured; $S$ is the standard deviation.

To describe the data distributions of IF_2_ and BC populations, the Kurtosis and Skewness were calculated using the formulae (3, 4) below:

3 $$\alpha =\frac{1}{n}\sum\nolimits_{i=1}^{n}{({{x}_{i}}-\bar{x}})_{{}}^{3}/{{S}^{3}}$$

4 $$\beta =\frac{1}{n}\sum\limits_{i=1}^{n}{({{x}_{i}}-\bar{x}})_{{}}^{4}/{{S}^{4}}-3$$

where $\alpha$ is Skewness; $\beta$ is Kurtosis; $S$ is standard deviation; $\bar{x}$ is mean of each sample; ${{x}_{i}}$ is the observed value of individual; *n* is the number of samples measured. When 0 < $\left| \alpha \right|$ or $\left| \beta \right|$ < 1, the data distribution was considered to be consistent with normal distribution.

One-way ANOVA analysis for each trait among environments and between parents HS46 and MAR was carried out following the formulas below (5–7): This ANOVA test is called the F-test statistic, and is typically identified with the letter: *F. F* = mean square between groups (MSA) divided by mean square within groups (MSE):

5 $$F=\frac{\text{MSA}}{\text{MSE}}=\frac{{}^{\text{SSA}}\!\!\diagup\!\!{}_{(k-1)}\;}{{}^{\text{SSE}}\!\!\diagup\!\!{}_{(kn-k)}\;}$$

6 $$\text{SSA}=\sum\limits_{i=1}^{k}{{{n}_{i}}{{({{{\bar{x}}}_{i}}-\bar{x})}^{2}}}$$

7 $$\text{SSE}=\sum\limits_{i=1}^{k}{\sum\limits_{j=1}^{{{n}_{i}}}{{{({{x}_{ij}}-{{{\bar{x}}}_{i}})}^{2}}}}$$

where MSA stands for between-group variance, MSE stands for within-group variance; *k* is the number of levels; *n* is the total number of samples measured; *n*_i_ is sample size of level *i*; ${{\bar{x}}_{i}}$ is the mean of level *i*; $\bar{x}$ is the mean of all samples measured; ${{x}_{ij}}$ is the observed value of No *j* of level *i*. If the value for *F* is greater than the critical *F* value at significance *p* = 0.05, it indicated that the difference between groups was significant. Otherwise, there is no difference.

### Genetic map

The genomic DNA from the mapping parents and populations were extracted and purified according to Paterson (1993). PCR was conducted by Kong’s method (Kong 2009). A total of 7825 pairs of molecular makers (SSRs, SRAPs, and RAPDs) were screened for polymorphism between the parents. The polymorphic primers were used to genotype the IF_2_ and BC populations, and 489 loci were obtained. Four hundred and eighty-nine markers in total were used to construct this genetic map. Segregation patterns for molecular marker data were scored and analyzed in JoinMap3.0 (Van Ooijen and Voorrips 2001). The Kosambi map function was used to convert recombination fractions to genetic distances. The linkage criteria was a significance level of *p* = 0.001. The minimum LOD score for linked markers was 3.0. Among 489 loci, 96 exhibited segregation distortion (*p* < 0.05). This linkage map contained a total of 388 molecular markers covering 15 chromosomes and 15 linkage groups with a total length of 1946.22 cm which accounts for 41.55% of the whole genome. It is a high density map with the average distance between each pair of markers being 5.03 cm; and it has a wide coverage in the cotton intra-specific RIL population. The genome of experimental materials consists of A and D sub-genome. Chromosomes 3, 5, 6, 7, 8, 9, and 13 belonged to sub-genome A and chromosomes 15, 16, 18, 21, 22, 23, and 25 belonged to sub-genome D.

### QTL software

QTLs for nine amino acids and their genetic effects including embryo additive and dominance main effects, maternal additive effects, as well as their environmental interaction effects, were analyzed with QTLNetwork-CL-2.0-seed software package (Yang et al. 2007), using the mixed linear model-based interval mapping with a 10 cm window size and a 1 cm walking speed. A 10 cm filtration window was used to distinguish the real QTLs on two adjacent test statistic peaks. One thousand permutation tests were performed on all traits in the combined data from two environments to calculate the critical *F* value at the 5% probability level. The genetic main effects of QTLs, the GE interaction effects, and corresponding *p* values were obtained by the Markov Chain Monte Carlo (MCMC) algorithm (Wang et al. 1994) for Gaussian mixed linear model via Gibbs sampling.

These QTLs were designated as ‘*q*’ followed by an abbreviation of the trait name, the chromosome, or linkage group location, and then the number assigned to the QTL related trait on a particular chromosome, based on the terminology of McCouch et al. (1997).

## Results

### Phenotypic analysis

#### IF_2_ population

Significant differences were detected between the parents HS46 and MAR for nine amino-acid contents in 2009 and 2010 (Table 1), HS46 were usually higher than those in MAR except for Gly and Pro contents in 2009. According to the absolute values (<1) of skewness and kurtosis, the distribution patterns for all nine amino-acid contents tested in *IF*_2_ population were normal and a wide variation for phenotypic value of these traits was found in both environments. Transgressive segregations in either direction were found for these amino-acid contents.

**Table 1:** Nine amino-acid contents (%) of cottonseed in IF_2_ population and parents in 2009 and 2010

Year	Trait	IF_2_						Parents	
		Mean	SD	Max	Min	Skew	Kurt	HS46	MAR
2009	Asp	3.56	0.19	4.10	3.11	0.16	−0.30	3.95a	3.90b
	Ser	1.49	0.05	1.61	1.35	−0.28	−0.23	1.53 A	1.50B
	Glu	8.46	0.44	9.57	7.33	−0.07	−0.40	9.23a	8.90b
	Gly	1.63	0.06	1.79	1.47	−0.06	−0.38	1.76a	1.73a
	Ala	1.52	0.05	1.66	1.37	−0.06	−0.28	1.64a	1.60b
	Tyr	1.02	0.06	1.21	0.86	−0.01	0.25	1.05 A	0.99B
	His	1.14	0.06	1.32	0.97	0.23	0.36	1.26a	1.20b
	Arg	4.87	0.32	5.60	3.94	−0.20	−0.39	5.33a	5.11b
	Pro	1.35	0.07	1.52	1.16	−0.32	−0.09	1.41a	1.37a
2010	Asp	3.67	0.12	4.12	3.31	0.46	0.83	3.76 A	3.57B
	Ser	1.53	0.03	1.62	1.42	−0.11	−0.11	1.55 A	1.51B
	Glu	8.96	0.28	9.60	8.18	−0.08	−0.43	9.17 A	8.80B
	Gly	1.70	0.04	1.82	1.57	−0.03	−0.02	1.73 A	1.69B
	Ala	1.56	0.04	1.67	1.44	0.04	0.00	1.59 A	1.53B
	Tyr	1.02	0.03	1.10	0.92	−0.17	−0.43	1.04 A	1.00B
	His	1.17	0.04	1.28	1.07	0.20	0.03	1.19 A	1.14B
	Arg	5.36	0.22	5.96	4.76	0.08	−0.13	5.50 A	5.15B
	Pro	1.40	0.04	1.50	1.29	−0.12	−0.41	1.43 A	1.38B

*Min* minimum, *Max* Maximum

a, b significance *P* = 0.05; A, B significance *P* = 0.01

#### BC population

The contents of nine amino acids were investigated in two reciprocal backcross (BC) populations which were derived from the same parents as in IF_2_ population (Table 2). Wide variation existed in the BC populations (BC_1_F_1_ and BC_2_F_1_) for these nine traits which they all showed an approximately normal distribution. There are significant differences in distributions between BC_1_F_1_ and BC_2_F_1_ populations for all traits, and the means of phenotypic values in BC_1_F_1_ were higher than those in BC_2_F_1_ in both years. Both backcross populations also showed varying distributions in 2009 and 2010, implying that these traits were subject to be affected by environments.

**Table 2:** Nine amino-acid contents (%) of cottonseed including parents and the backcross population (BC_1_F_1_ and BC_2_F_1_) of RILs in 2009 and 2010

Year	Trait	Parents		BC (BILS × P_1_)				BC (BILS × P_2_)			
		HS46	MAR	Mean	SD	Min	Max	Mean	SD	Min	Max
2009	Asp	3.95a	3.90b	3.81	0.20	3.29	4.48	3.76	0.23	3.30	4.45
	Ser	1.53A	1.50B	1.52	0.06	1.36	1.70	1.48	0.07	1.32	1.69
	Glu	9.23a	8.90b	8.74	0.42	7.39	9.70	8.53	0.47	7.39	9.74
	Gly	1.76a	1.73a	1.70	0.07	1.51	1.89	1.68	0.07	1.50	1.86
	Ala	1.64a	1.60b	1.59	0.06	1.44	1.77	1.57	0.07	1.43	1.76
	Tyr	1.05A	0.99B	1.01	0.05	0.88	1.14	0.98	0.06	0.83	1.18
	His	1.26a	1.20b	1.21	0.07	1.03	1.40	1.20	0.07	1.04	1.42
	Arg	5.33a	5.11b	5.23	0.33	4.30	6.16	5.11	0.39	4.09	6.08
	Pro	1.41a	1.37a	1.38	0.06	1.22	1.55	1.35	0.07	1.16	1.56
2010	Asp	3.76A	3.57B	3.75	0.12	3.50	4.22	3.67	0.12	3.28	4.02
	Ser	1.55A	1.51B	1.52	0.03	1.44	1.65	1.50	0.04	1.37	1.59
	Glu	9.17A	8.80B	9.13	0.27	8.30	9.99	8.98	0.26	7.99	9.60
	Gly	1.73A	1.67B	1.71	0.04	1.61	1.84	1.68	0.04	1.53	1.78
	Ala	1.59A	1.53B	1.60	0.03	1.52	1.72	1.58	0.03	1.44	1.67
	Tyr	1.04A	1.00B	1.04	0.03	0.91	1.12	1.02	0.03	0.89	1.10
	His	1.19A	1.14B	1.19	0.03	1.11	1.31	1.17	0.03	1.06	1.26
	Arg	5.50A	5.15B	5.48	0.21	4.79	6.15	5.39	0.22	4.58	5.95
	Pro	1.43A	1.38B	1.42	0.04	1.32	1.54	1.41	0.04	1.26	1.51

*Min* minimum, *Max* Maximum

a, b significance *p* = 0.05; A, B significance *p* = 0.01

**Fig. 1:**
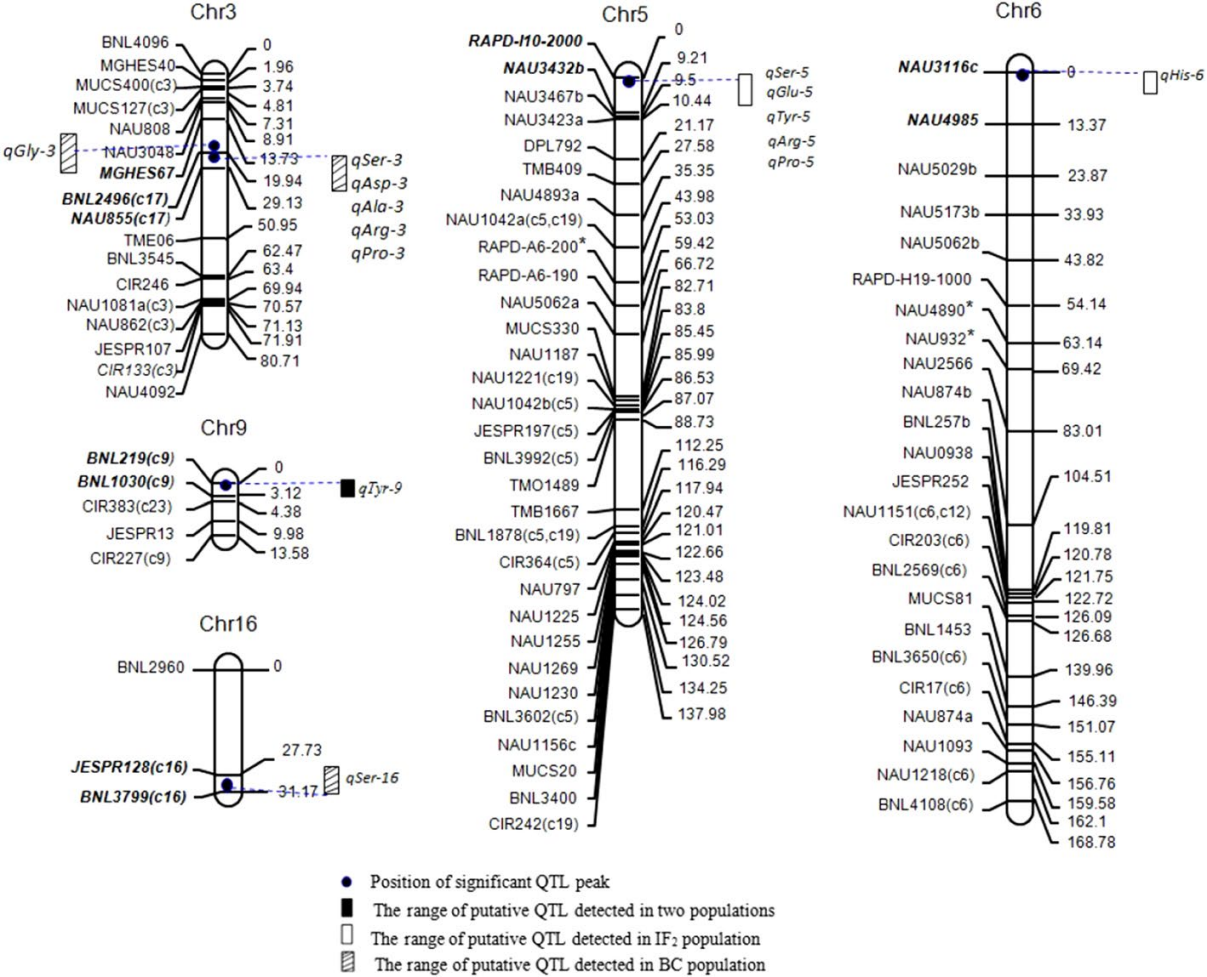

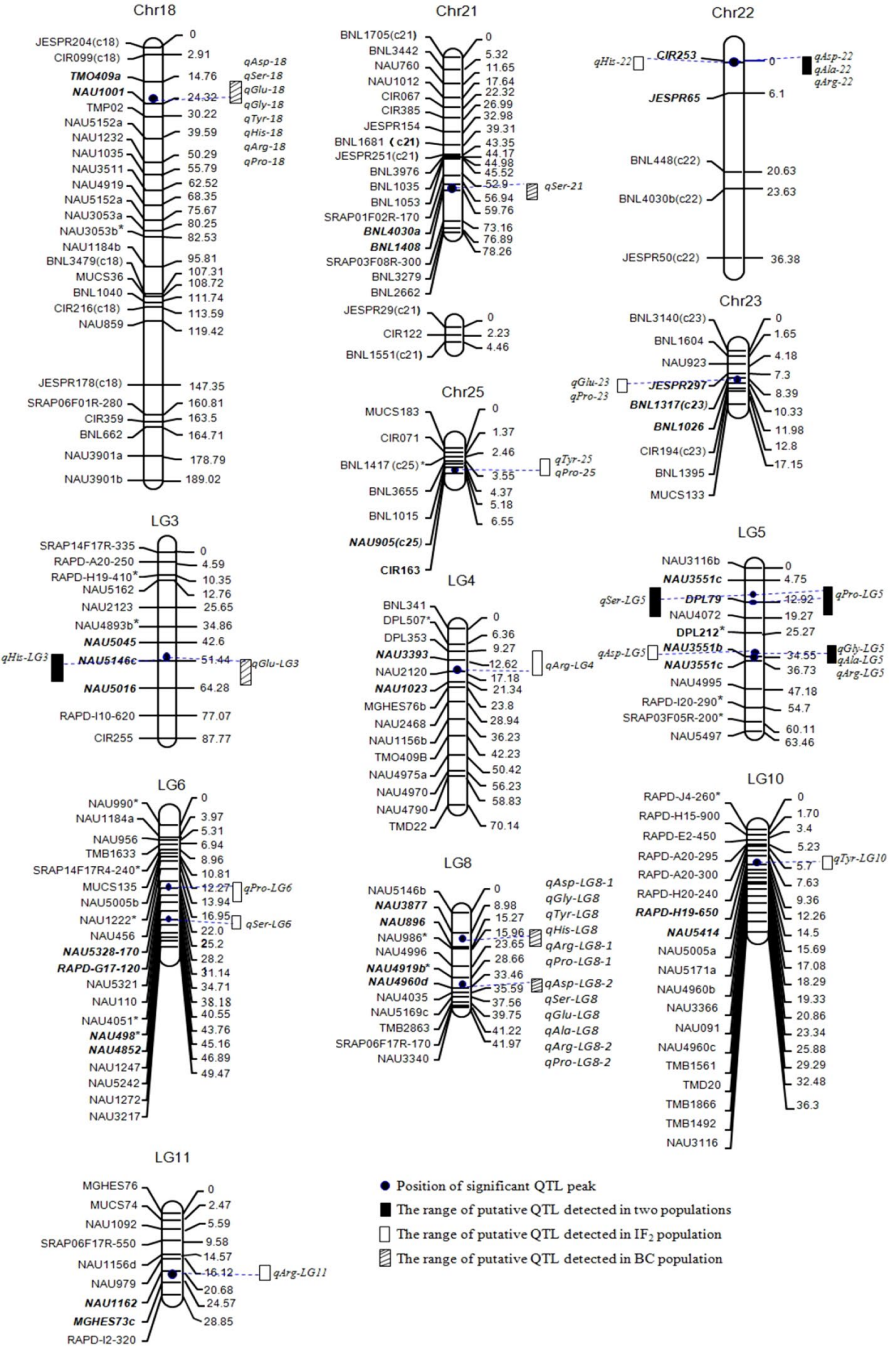
Position of QTL in IF_2_ and BC populations

### QTL analysis

Significant QTLs for the nine amino acids in IF_2_ and BC populations were listed in Tables 3, 4 and Fig. 1. Among 56 QTLs found in present experiment for these traits, the direction of embryo additive main effect (*a* ^*e*^) and maternal additive main effect (*a* ^*m*^) was opposite for most of the QTLs, which showed that the parents HS46 and MAR both had the favorable alleles from embryo genome or maternal plant genome (Tables 5, 6). For most QTLs with over 5% of phenotypic variation, the genetic effects from embryo genome were larger than those from maternal plant genome. Among 56 QTLs, 50 QTLs had embryo additive main effects and 45 QTLs had significant maternal additive main effects, suggesting that the genetic effect from both of embryo and maternal nuclear genomes was important. Thirty-five QTLs had significant embryo dominance main effects (*d* ^*e*^), implying heterosis in these loci. Thirty-one QTLs had significant environment interaction effects, indicating that the influence of environmental factors on the expression of some QTLs located in different genetic systems could not be ignored for these nine amino acids .

**Table 3:** Identification and contribution of QTL for nine amino acids of cottonseed in *IF*_2_ population

Trait	QTL	Chr/LG	Mark interval	Position	Range	*R* ^2^ (%)
Asp	*qAsp-22*	22	CIR253-JESPR65	0.0	0.0–3.0	26.3
	*qAsp-LG5*	LG5	DPL212-NAU3551b	34.3	30.3–35.5	21.5
Ser	*qSer-5*	5	RAPD-I10-2000-NAU3432b	3.0	0.0–6.0	6.6
	*qSer-LG5*	LG5	DPL79-NAU4072	14.9	8.8–15.9	7.6
	*qSer-LG6*	LG6	NAU498*-NAU4852	39.2	37.7–40.5	38.3
Glu	*qGlu-5*	5	RAPD-I10-2000-NAU3432b	0.0	0.0–4.0	8.7
	*qGlu-23*	23	BNL1317-BNL1026	9.4	6.2–11.3	51.5
Gly	*qGly-LG5*	LG5	NAU3551b-NAU3551c	34.5	31.3–36.5	19.1
Ala	*qAla-22*	22	CIR253-JESPR65	0.0	0.0–3.0	36.2
	*qAla-LG5*	LG5	NAU3551b-NAU3551c	34.5	31.3–36.5	17.4
Tyr	*qTyr-5*	5	RAPD-I10-2000-NAU3432b	4.0	0.0–5.0	26.9
	*qTyr-9*	9	BNL219-BNL1030	0.0	0.0–3.0	1.0
	*qTyr-25*	25	NAU905-CIR163	5.2	3.5–6.2	9.9
	*qTyr-LG10*	LG10	RAPD-H19-650-NAU5414	9.4	8.6–11.4	4.2
His	*qHis-6*	6	NAU3116c-NAU4985	0.0	0.0–4.0	19.8
	*qHis-22*	22	CIR253-JESPR65	0.0	0.0–3.0	23.6
	*qHis-LG3*	LG3	NAU5045-NAU5146c	50.6	47.6–62.4	9.3
Arg	*qArg-5*	5	RAPD-I10-2000-NAU3432b	3.0	0.0–6.0	1.4
	*qArg-22*	22	CIR253-JESPR65	0.0	0.0–3.0	15.6
	*qArg-LG4*	LG4	NAU3393-NAU1023	15.6	10.3–17.6	38.7
	*qArg-LG5*	LG5	DPL212-NAU3551b	34.3	30.3–36.5	27.2
	*qArg-LG11*	LG11	NAU1162-MGHES73c	20.7	19.1–22.7	0.9
Pro	*qPro-5*	5	RAPD-I10-2000-NAU3432b	3.0	0.0–5.0	10.0
	*qPro-23*	23	JESPR297-BNL1317	8.3	6.2–10.3	7.9
	*qPro-25*	25	NAU905-CIR163	5.2	4.4–6.2	36.5
	*qPro-LG5*	LG5	NAU3551c-DPL79*	9.8	8.8–16.9	3.4
	*qPro-LG6*	LG6	NAU5328-170-RAPD-G17-120	25.0	23.0-27.2	0.5

Chr/LG represents chromosome (Chr) or a particular linkage group (LG); position represents the position of *F* peak value for QTL; *R* ^2^ represents the phenotypic variations explained by single QTL; overstriking QTLs show the common QTL in two populations

**Table 4:** Identification and contribution of QTL for nine amino acids of cottonseed in BC population

Trait	QTL	Chr/LG	Mark interval	Position	Range	*R* ^2^ (%)
Asp	*qAsp-3*	3	BNL2496-NAU855	21.9	15.7–27.9	16.5
	*qAsp-18*	18	TMO409a-NAU1001	16.8	13.9–21.8	4.1
	*qAsp-22*	22	CIR253-JESPR65	0.0	0.0–3.0	12.3
	*qAsp-LG8-1*	LG8	NAU3877-NAU896	13.0	10.0-15.3	1.5
	*qAsp-LG8-2*	LG8	NAU4919b*-NAU4960d	31.7	29.7–32.7	5.8
Ser	*qSer-3*	3	BNL2496-NAU855	22.9	17.7–26.9	0.8
	*qSer-16*	16	JESPR128-BNL3799	30.7	27.7–30.7	1.7
	*qSer-18*	18	TMO409a-NAU1001	14.8	12.9–17.8	0.3
	*qSer-21*	21	BNL4030a-BNL1408	58.9	56.5–63.8	0.6
	*qSer-LG5*	LG5	DPL79-NAU4072	14.9	8.8–15.9	16.6
	*qSer-LG8*	LG8	NAU4919b*-NAU4960d	29.7	28.6–31.7	44.4
Glu	*qGlu-18*	18	TMO409a-NAU1001	14.8	12.9–17.8	2.1
	*qGlu-LG3*	LG3	NAU5146c- NAU5016	51.4	49.6–55.4	15.0
	*qGlu-LG8*	LG8	NAU4919b*-NAU4960d	30.7	29.7–32.7	46.1
Gly	*qGly-3*	3	MGHES67-BNL2496	18.7	14.7–26.9	11.8
	*qGly-18*	18	TMO409a-NAU1001	15.8	11.9–18.8	10.2
	*qGly-LG5*	LG5	NAU3551b-NAU3551c	34.5	31.3–36.5	6.7
	*qGly-LG8*	LG8	NAU3877-NAU896	15.0	13.0–16.0	3.6
Ala	*qAla-3*	3	BNL2496-NAU855	21.9	14.7–28.9	11.4
	*qAla-22*	22	CIR253-JESPR65	0.0	0.0–3.0	11.4
	*qAla-LG5*	LG5	NAU3551b-NAU3551c	34.5	31.3–36.5	20.3
	*qAla-LG8*	LG8	NAU4919b*-NAU4960d	30.7	29.7–31.7	12.0
Tyr	*qTyr-9*	9	BNL219-BNL1030	0.0	0.0–3.0	4.8
	*qTyr-18*	18	TMO409-S-NAU1001	14.8	11.9–18.8	0.2
	*qTyr-LG8*	LG8	NAU3877-NAU896	14	11.0–16.0	10.5
His	*qHis-18*	18	TMO409a-NAU1001	17.8	14.8–21.8	3.4
	*qHis-LG3*	LG3	NAU5045-NAU5146c	50.6	47.6–62.4	13.0
	*qHis-LG8*	LG8	NAU3877-NAU896	13.0	10.0–15.0	2.7
Arg	*qArg-3*	3	BNL2496-NAU855	21.9	17.7–26.9	6.8
	*qArg-18*	18	TMO409a-NAU1001	15.8	12.9–20.8	1.9
	*qArg-22*	22	CIR253-JESPR65	0.0	0.0–2.0	3.0
	*qArg-LG5*	LG5	DPL212-NAU3551b	34.3	30.3–36.5	11.7
	*qArg-LG8-1*	LG8	NAU3877-NAU896	13.0	10.0–15.0	3.8
	*qArg-LG8-2*	LG8	NAU4919b*-NAU4960d	30.7	28.7–31.7	38.9
Pro	*qPro-3*	3	BNL2496-NAU855	20.9	16.7–25.9	5.3
	*qPro-18*	18	TMO409a-NAU1001	14.8	12.9–17.8	1.9
	*qPro-LG5*	LG5	NAU3551c-DPL79*	9.8	8.8–16.9	12.4
	*qPro-LG8-1*	LG8	NAU3877-NAU896	14.0	11.0–16.0	3.3
	*qPro-LG8-2*	LG8	NAU4919b*-NAU4960d	30.7	28.7–31.7	38.3

Chr /LG represents chromosome (Chr) or a particular linkage group (LG); position represents the position of *F* peak value for QTL; *R* ^2^ represents the phenotypic variations explained by single QTL

**Table 5:** Genetic main effects and GE interaction effects from the QTLs for nine amino acids of cottonseed across environments in *IF*_2_ populations

Trait	QTL	*a* ^*e*^	*d* ^*e*^	*a* ^*m*^	*a* ^*e*^ *E*_*1*_	*a* ^*e*^ *E*_*2*_	*d* ^*e*^ *E*_*1*_	*d* ^*e*^ *E*_*2*_	*a* ^*m*^ *E*_*1*_	*a* ^*m*^ *E*_*2*_
Asp	*qAsp-22*	−0.56	−0.14	−0.92*	−0.01	1.77**	0.90	−0.90	0.01	−1.73**
	*qAsp-LG5*	−3.89**	−0.41	2.64**	−0.02	−0.50	−0.62	0.61	0.02	0.50
Ser	*qSer-5*	−0.56**	−1.41**	0.33*	−0.05	0.02	−0.62*	0.61*	0.06	−0.02
	*qSer-LG5*	−0.10	−0.47*	1.07**	−0.40*	−0.11	−0.35	0.34	0.40*	0.12
	*qSer-LG6*	−0.80**	0.03	0.80**	−0.75**	0.93**	0.03	−0.03	0.72**	−0.93**
Glu	*qGlu-5*	−5.36**	−8.77**	3.52**	−1.29	1.48	−2.72	2.73	1.32	−1.46
	*qGlu-23*	5.02**	−5.46**	−1.64	2.02	−5.12**	−1.86	1.84	−2.06	5.05**
Gly	*qGly-LG5*	−1.37**	−0.16	0.94**	−0.02	−0.05	−0.12	0.11	0.01	0.05
Ala	*qAla-22*	−0.45**	−0.23	0.07	−0.04	0.67**	0.05	−0.05	0.05	−0.66**
	*qAla-LG5*	−1.21**	−0.20	0.92**	−0.03	−0.12	−0.11	0.11	0.03	0.12
Tyr	*qTyr-5*	−1.17**	−1.07**	0.74**	−1.18**	1.31**	−0.06	0.06	1.14**	−1.46**
	*qTyr-9*	0.37**	−0.93**	−0.59**	0.01	0.16	−0.53*	0.55**	−0.01	−0.16
	*qTyr-25*	0.97**	0.06	−0.74**	0.69**	−0.86**	0.26	−0.26	−0.68**	0.81**
	*qTyr-LG10*	0.29**	0.21	−0.99**	0.60**	−0.39*	0.00	0.00	−0.62**	0.38**
His	*qHis-6*	0.45**	0.95**	−0.04	−0.06	0.00	0.76**	−0.77**	0.06	0.00
	*qHis-22*	−0.11	−0.12	−0.31*	−0.01	0.60**	0.05	−0.05	0.01	−0.59**
	*qHis-LG3*	0.63**	−0.12	0.02	−0.24	−0.04	−0.13	0.13	0.24	0.04
Arg	*qArg-5*	−4.12**	−6.41**	2.82**	−1.36	1.62	0.00	0.00	1.45	−1.64
	*qArg-22*	−1.43*	−1.30	−0.83	−0.03	3.12**	2.55	−2.58	0.03	−2.88**
	*qArg-LG4*	−12.94**	−16.51**	8.50**	−0.03	−0.03	−7.18**	6.96**	0.03	0.03
	*qArg-LG5*	−6.43**	−1.21	4.80**	0.01	−0.82	−1.08	1.09	−0.01	0.85
	*qArg-LG11*	1.24*	3.11*	−4.4**	0.01	1.44	0.19	−0.19	−0.01	−1.43
Pro	*qPro-5*	−0.84**	−1.58**	0.44**	−0.52*	0.57**	−0.69*	0.68*	0.57	−0.58**
	*qPro-23*	0.64**	−0.72**	0.06	0.06	−0.69**	−0.31	0.31	−0.06	0.74**
	*qPro-25*	1.33**	0.21	−0.95**	1.07*	−1.16**	0.39	−0.4	−1.09**	1.21**
	*qPro-LG5*	−0.56**	−0.50	1.62**	−0.39	−0.12	0.00	0.00	0.37	0.13
	*qPro-LG6*	0.21	−0.83**	0.49**	−0.20	0.02	−0.01	0.01	0.20	−0.02

The negative sign (−) before a genetic effect represent the allele from MARCABUCAG8US-1-88 increasing the value of the trait

*a* ^*e*^ embryo additive main effect, *d* ^*e*^ embryo dominance main effect, *a* ^*m*^ maternal additive main effect, *a* ^*e*^E_1_ *and a* ^*e*^E_2_ embryo additive interaction effects in environment 1 and environment 2, *d* ^*e*^E_1_ *and d* ^*e*^E_2_ embryo dominance interaction effects in environment 1 and environment 2, *a* ^*m*^E_1_ *and a* ^*m*^E_2_ maternal additive interaction effects in environment 1 and environment 2, respectively

*p < 0.05; **p < 0.01

**Table 6:** Genetic main effects and GE interaction effects from the QTL for nine amino acids of cottonseed across environments in BC population

Trait	QTL	*a* ^*e*^	*d* ^*e*^	*a* ^*m*^	*a* ^*e*^ *E*_*1*_	*a* ^*e*^ *E*_*2*_	*d* ^*e*^ *E*_*1*_	*d* ^*e*^ *E*_*2*_	*a* ^*m*^ *E*_*1*_	*a* ^*m*^ *E*_*2*_
Asp	*qAsp-3*	1.93**	−0.47	−1.94**	1.51*	0.18	0.04	−0.05	−1.54**	−0.18
	*qAsp-18*	1.75**	2.89**	−1.77	0.44	0.17	0.09	−0.09	−0.45	−0.17
	*qAsp-22*	−6.30**	−0.35	6.20*	1.55**	0.18	−0.21	0.21	−1.52*	−0.18
	*qAsp-LG8-1*	1.06**	4.02**	−1.08**	0.06	0.16	0.09	−0.09	−0.09	−0.16
	*qAsp-LG8-2*	−2.11**	−0.47	2.06**	2.04*	0.19	−0.03	0.03	−2.05*	−0.19
Ser	*qSer-3*	−0.27	0.08	2.69**	0.34	0.24	−0.04	0.04	−0.34	−0.24
	*qSer-16*	4.02**	0.31	−0.40*	−0.22	−0.10	0.03	−0.03	0.22	0.10
	*qSer-18*	−1.78**	1.02**	1.71**	0.08	0.03	0.27	−0.26	−0.08	−0.01
	*qSer-21*	2.37**	0.22	−2.43	0.11	0.30	0.00	0.00	−0.10	−0.31
	*qSer-LG5*	−3.73**	0.61**	3.78**	−0.37	−0.37*	0.69**	−0.68	0.36	0.37*
	*qSer-LG8*	7.00**	−0.49*	−7.20*	0.95**	−0.14	−0.17	0.17	−0.96	0.16
Glu	*qGlu-18*	3.10**	5.67**	−3.15**	0.04	0.32	0.03	−0.03	−0.04	−0.32
	*qGlu-LG3*	−4.78**	1.11	4.84**	−1.75	−3.11**	−0.03	0.03	1.74	3.18**
	*qGlu-LG8*	2.96**	−3.18*	−3.05**	6.00**	0.04	0.00	0.00	−6.01**	−0.10
Gly	*qGly-3*	3.76**	−0.17	−0.38	0.03	0.44*	−0.01	0.01	−0.03	−0.42*
	*qGly-18*	−0.94**	0.97**	0.93*	0.10	0.02	0.03	−0.03	−0.11	−0.03
	*qGly-LG5*	4.96**	0.36	−4.56**	−0.44	−0.19	0.64*	−0.64*	0.46	0.20
	*qGly-LG8*	5.54**	0.59**	−5.62**	0.13	0.24	0.52	−0.50	−0.13	−0.24
Ala	*qAla-3*	5.57**	−0.06	−5.62**	0.14	0.31*	0.00	0.00	−0.15	−0.32*
	*qAla-22*	3.90**	0.01	−3.94**	0.15	0.39*	−0.07	0.07	−0.15	−0.38*
	*qAla-LG5*	−3.20**	0.42*	3.24**	−0.53*	−0.15	0.55*	−0.54*	0.52*	0.15
	*qAla-LG8*	−5.82**	−0.33	5.69**	0.14	0.46*	0.00	0.00	−0.14	−0.40
Tyr	*qTyr-9*	−19.26**	−0.07	18.90**	0.43*	0.03	0.03	−0.03	−0.41*	−0.03
	*qTyr-18*	37.09**	0.64**	−37.71**	0.02	0.01	−0.01	0.01	−0.01	−0.01
	*qTyr-LG8*	−0.76*	0.45*	0.75*	0.38*	0.03	0.77**	−0.76**	−0.38*	−0.03
His	*qHis-18*	49.15**	0.97**	−49.8**	0.02	0.06	0.01	−0.01	−0.02	−0.06
	*qHis-LG3*	−7.61**	−0.36	7.18**	−0.40*	−0.19	−0.01	0.01	0.39*	0.21
	*qHis-LG8*	43.15**	1.00**	−44.02**	0.22	0.08	0.32	−0.31	−0.22	−0.08
Arg	*qArg-3*	−1.82*	−0.76	1.79**	3.03	0.18	−0.02	0.02	−3.03**	−0.16
	*qArg-18*	2.63**	5.74**	−2.67**	0.03	−0.23	1.68	−1.62	−0.03	0.23
	*qArg-22*	1.17**	−0.62	−1.19**	1.31	1.31	−3.33*	3.29*	−1.30	−1.34
	*qArg-LG5*	−2.35**	4.21**	2.37**	−3.20**	−0.09	3.95**	−3.91**	3.17**	0.09
	*qArg-LG8-1*	−3.75**	4.42**	3.70**	0.07	0.61	2.78	−2.83	−0.07	−0.67
	*qArg-LG8-2*	4.85**	−2.66*	−4.92**	7.12**	−2.17	−0.01	0.01	−7.14**	2.22
Pro	*qPro-3*	0.59*	0.09	−0.59*	0.40*	0.14	−0.05	0.05	−0.40*	−0.14
	*qPro-18*	0.51*	1.03**	−0.52*	0.05	0.03	0.23	−0.23	−0.05	−0.03
	*qPro-LG5*	−3.43**	0.50*	3.47**	−0.19	−0.52**	0.90**	−0.90**	0.19	0.53**
	*qPro-LG8-1*	3.13**	0.76**	−3.20**	0.07	0.17	0.62*	−0.63*	−0.07	−0.17
	*qPro-LG8-2*	−6.45**	−0.70**	6.32**	1.40**	−0.10	0.00	0.00	−1.42**	0.11

The negative sign (−) before a genetic effect represents the allele from MARCABUCAG8US-1-88 increasing the value of the trait.

*a* ^*e*^, embryo additive main effect, *d* ^*e*^ EMBRYO dominance main effect, *a* ^*m*^ maternal additive main effect, *a* ^*e*^ *E*_*1*_ *and a* ^*e*^ *E*_*2*_ embryo additive interaction effects in environment 1 and environment 2, *d* ^*e*^ *E*_*1*_ *and d* ^*e*^ *E*_*2*_ embryo dominance interaction effects in environment 1 and environment 2, *amE1 and amE2* maternal additive interaction effects in environment 1 and environment 2, respectively.

*p < 0.05; **p < 0.01

#### Common QTL

Common significant QTLs for the traits except for Glu were detected in these two different kinds of populations. A total of ten QTLs associated with eight quality traits were found, and the significance of the genetic effects of these QTLs varied in different populations. Seven of these ten QTLs had high contribution (more than 5%) in both populations.

For Asp, one common QTL, namely, *qAsp-22*, was located between makers CIR253 and JESPR65 on chromosome 22 of D sub-genome (D22). It could explain phenotypic variation of 26.3 and 12.3% in IF_2_ and BC populations, respectively. *qAsp-22* was a major QTL according to the research by Yang et al.(2008). Significant maternal additive effect (*a* ^*m*^) and maternal additive × environment interaction effect (*a* ^*m*^ *E*) were detected in both populations (Tables 5, 6), and the genetic effects from *qAsp-22* in maternal genome were larger than those from embryo genome, implying that this locus in maternal plant genome was more important for the performance of Asp in cottonseed.

Among all QTLs of Ser content, only one QTL (*qSer-LG5*) was detected in both populations. It was mapped on linkage group 5. It had high contribution (over 5%) and the largest maternal additive main effect in both populations. The embryo additive effect was only found in BC populations, suggesting that it varied with population. These results showed that the additive main genetic effect from the maternal plant genome was more important in affecting the performance of Ser content.

*qGly-LG5* of Gly content, located between markers NAU3551b and NAU3551c on the linkage group 5, was the only common QTL. It could explain phenotypic variation of 19.1% in IF_2_ population, whereas in the BC populations, it could explain a lower phenotypic variation (6.7%). Even so, this QTL still belonged to a major QTL in both populations. Significant *a* ^*e*^ and *a* ^*m*^ were simultaneously found, suggesting that the additive main effects from either embryo genome or maternal genome were important.

There were two common QTLs, namely *qAla-22 and qAla-LG5* in both populations for Ala content of cottonseeds. *qAla-22* in IF_2_ population was mapped at the interval between markers CIR253 and JESPR65 on chromosome D22. Significant embryo additive main effect was found in both populations, implying that the expression of the QTL located on the embryo chromosome was more important for the performance of Ala content. In addition, significant QTL× environment interaction effects such as embryo and maternal additive interaction effects in environment 2 (*a* ^*e*^ *E*_*2*_ and *a* ^*m*^ *E*_*2*_) were detected in both populations, suggesting that this QTL was easily subjected by the environmental influence. *qAla-LG5* located on linkage group 5 had high contribution to Ala content, which could explain a phenotypic variation of 17.4% in the IF_2_ population and 20.3% in the BC populations, respectively. Since there were significant *a* ^*e*^ and *a* ^*m*^ in both populations, the performance of Ala content in cottonseeds was mainly controlled by the additive main effect of this QTL on this locus.

One significant QTL for Tyr content, namely *qTyr-9*, was detected in both populations. It was located at the interval between the makers BNL219 and BNL1030 on chromosome A9. The additive main effects including *a* ^*e*^ and *a* ^*m*^ from embryo genome and maternal plant genome, respectively, were significant in both populations. It showed that this QTL was still useful for improvement of Tyr content, although it had contributed in a lower proportion to phenotypic variation.

In addition, only one QTL for His content, *qHis-LG3*, was significant in IF_2_ and BC populations. It was identified in the interval between NAU5045 and NAU5146b on linkage group 3. Its contribution to phenotypic variation was over 5% and with significant *a* ^*e*^ in both populations. The notable *a* ^*m*^ was only found in BC populations, while it was not significant in IF_2_ population. These results, therefore, indicated that it was a major QTL for improving His content and the additive main effects from embryo genome were more important than those from maternal plant genome.

Two significant QTLs for Arg content, namely, *qArg-22 and qArg-LG5*, were identified in IF_2_ and BC populations. *qArg-22* located between the markers CIR253 and JESPR65 on chromosome D22 had a higher contribution (*R* ^2^ = 15.6%) in IF_2_ population than that in BC populations. Significant *a* ^*e*^ could be simultaneously found in both populations, while other genetic effects such as *a* ^*m*^, *a* ^*e*^ *E*_*2*_ and *d* ^*e*^ *E*_*1*_ were only found in one of the populations. These results indicated that the additive main effect from this QTL located on embryo genome was more important than those from maternal plant genome. *qArg-LG5*, located on the linkage group 5 had significant *a* ^*e*^ and *a* ^*m*^ in both populations, implying that the genetic effects from the embryo genome and from the maternal plant genome were both important for affecting Arg content in cottonseeds. The genetic effect of this QTL accounted for 27.2 and 11.7% of phenotypic variation in IF_2_ population and BC population, respectively. This result showed that this was a major QTL.

There was only one common QTL (*qPro-LG5*) detected in both population for Pro content. It was located at the interval between the markers NAU3551c and DPL79* on linkage group 5. The significant *a* ^*e*^ and *a* ^*m*^ could be found in these two populations, whereas other genetic effects such as *d* ^*e*^, *a* ^*e*^ *E*_*2*_, *d* ^*e*^ *E*_*1*_, *d* ^*e*^ *E*_*2*_, and *a* ^*m*^ *E*_*2*_ varied with different populations. The *a* ^*m*^ was larger than the *a* ^*e*^, implying that the additive effect from maternal plant genome was more important. Significant *a* ^*e*^ *E*_*2*_, *d* ^*e*^ *E*_*1*_, *d* ^*e*^ *E*_*2*_, and *a* ^*m*^ *E*_*2*_ were only found in the BC populations, which suggested that the environmental factors should be considered as well.

#### Individual QTL

Many significant QTLs were found in only one of these two populations (Tables 3, 4). Overall, there were more QTLs in BC populations than those in IF_2_ population. However, the contributions for most QTLs were higher in IF_2_ population than those in BC populations. Some QTLs which were only detected in a single population could be important for the performance of corresponding traits, as they would suggest the following examples:

*qAsp-3* of Asp content was mapped at the interval between markers BNL2496 and NAU855 on chromosome A3 in BC populations, which could explain 16.5% of the phenotypic variation. The genetic effects of this QTL including *a* ^*e*^, *a* ^*m*^, and their interaction effects in environment 1 were all significant. The direction of these genetic effects from this QTL was negative from the maternal genome, while they were positive from the embryo genome.

Three QTLs for Ser content in IF_2_ population and six in BC populations. *qSer-5* for Ser content in IF_2_ population, located at the interval between markers RAPD-I10-2000 and NAU3432b on chromosome A5, had the largest dominance main effect. Moreover, they were found to have significant dominance interaction effects in two environments. These results showed strong heterosis possibility occurring in this locus. *qSer-LG8* for Ser content in BC populations was mapped at an interval between markers NAU4919b* and NAU4960d on linkage group 8. The *a* ^*e*^, *d* ^*e*^, *a* ^*m*^, and embryo additive interaction effect in environment 1 (*a* ^*e*^ *E*_*1*_) from this QTL were significant. The genetic effects of this QTL accounted for 44.4% of phenotypic variation. It had the largest *a* ^*e*^ and *a* ^*m*^, and *a* ^*e*^ was positive, while *a* ^*m*^ was negative.

Two QTLs for Glu content were detected in IF_2_ population and three in BC populations. With 8.7% of the phenotypic variation in IF_2_ population, *qGlu-5* was located at the interval between markers RAPD-I10-2000 and NAU3432b on chromosome A5. It was found to have only significant genetic main effects including *a* ^*e*^, *d* ^*e*^, *and a* ^*m*^, suggesting that the expression of this QTL was very stable through the 2 years. It had the largest *a* ^*e*^, *d* ^*e*^, and *a* ^*m*^, and the allele in this locus from MAR could increase 5.36% and 8.77% by *a* ^*e*^ and *d* ^*e*^, respectively, while that from HS46 could increase 3.52% by *a* ^*m*^. Another QTL for Glu content, *qGlu-23*, in IF_2_ was mapped at the interval between makers BNL1317 and BNL1026 on chromosome D23. It could explain 51.5% of the phenotypic variation. Significant *a* ^*e*^, *d* ^*e*^, embryo additive interaction, and maternal additive interaction in environment 2 (*a* ^*e*^ *E*_*2*_ and *a* ^*m*^ *E*_*2*_) were found, implying that the genetic effects from embryo genome were more important for the performance of Glu content in this locus. *qGlu-LG3* in BC populations located on linkage group 3 could explain 15.0% of the phenotypic variation. It had the largest *a* ^*e*^ and *a* ^*m*^, which showed that the genetic effects from these two different genomes were important for affecting the performance of Glu content. Significant *a* ^*e*^E_2_ and *a* ^*m*^E_2_ were found as well, implying that environmental factor could not be ignored.

Only one QTL for Gly content was identified in the IF_2_ population, while four were found in the BC populations. In the BC populations, *qGly-3* with the highest contribution to phenotypic variation, was identified on chromosome A3. Significant *a* ^*e*^, *a* ^*e*^ *E*_*2*_, and *a* ^*m*^ *E*_*2*_ were simultaneously detected, implying that Gly content was mainly affected by the genetic effects from embryo genome.

Among all four QTLs for Ala content in the BC populations, *qAla-3*, with 11.4% of the phenotypic variation, was mapped in the interval between makers BNL2496 and NAU855 on chromosome A3. It was found to have significant *a* ^*e*^, *a* ^*m*^, *a* ^*e*^ *E*_*2*_, and *a* ^*m*^E_2_, which showed that additive effects from embryo and maternal plant genome were both important for the performance of Ala content at this locus.

A total of six QTLs were detected for Tyr content in both populations. *qTyr-5* in IF_2_ population, located at the interval between markers RAPD-I10-2000 and NAU3432b on Chromosome A5, could explain phenotypic variation of 26.9%. Significant genetic effects except for embryo dominance environmental interaction (*d* ^*e*^ *E*_*1*_ and *d* ^*e*^ *E*_*2*_) were all detected. It had the largest *a* ^*e*^ and *d* ^*e*^. Moreover, these kinds of genetic effects were both larger than *a* ^*m*^, suggesting that the genetic effects from embryo genome were more important than those from maternal plant genome in affecting Tyr content in this locus. In addition, environmental factor was also vital for the expression of this QTL.

Among all five QTLs for His content in both populations, *qHis-6* in the IF_2_ population, located in the interval between NAU3116c and NAU4985 on chromosome A6, could explain phenotypic variation of 19.8%. Significant *a* ^*e*^, *d* ^*e*^, *d* ^*e*^ *E*_1_, and *d* ^*e*^ *E*_2_ were found, showing that the performance of His content was mainly affected by the genetic effects from embryo genome in this locus. It had the largest *d* ^*e*^, implying that strong heterosis existed at this locus.

One of QTLs in the IF_2_ population, namely, *qArg-LG4* for Arg content, identified on linkage group 4, could explain phenotypic variation of 38.4%. It was found to have significant *a* ^*e*^, *d* ^*e*^, *a* ^*m*^, *d* ^*e*^E_1_ and *d* ^*e*^E_2_. These genetic effects were the largest among all the QTLs detected for Arg content. *a* ^*e*^ (−12.94**) and *d* ^*e*^ (−16.51**) were negative, which showed that the allele from parent MAR could increase Arg content of 12.94 and 16.51% by *a* ^*e*^ *and d* ^*e*^, respectively. *a* ^*m*^ (8.50**) was positive, which indicated that the allele from the HS46 could also increase Arg content of 8.5% by *a* ^*m*^. These results above showed that the genetic effects from embryo and maternal genomes were both vital for the performance of Arg content. In the BC populations, one QTL, *qArg-LG8-2*, located on linkage group 8, had the largest *a* ^*e*^ and *a* ^*m*^. Moreover, *a* ^*m*^ was the largest among all the genetic main effects, implying that this genetic effect from maternal plant genome could be more important in affecting Arg content. Significant embryo additive and maternal additive interaction effects in environment 1 (*a* ^*e*^E_1_ and *a* ^*m*^E_1_) were found as well, which showed that the expression of this QTL was easily subjected to environmental effects.

In the IF_2_ population, a total of five QTLs of Pro content were detected and three of them had over 5% of phenotypic variation. Significant *a* ^*e*^ and *a* ^*m*^ were found for four QTLs. *qPro-25* with the largest *a* ^*e*^ was mapped at the interval between markers NAU905 and CIR163 on chromosome D25. This QTL could explain a phenotypic variation of 36.5%. Among the five QTLs, the *a* ^*m*^ (−0.95**) of this QTL was the second largest, suggesting that the additive main effect from maternal plant genome at this locus was also important in affecting Pro content. In addition, significant embryo additive and maternal additive interaction effects were simultaneously found, suggesting that the expression of this QTL was easily influenced by environmental factors. In the BC population, a total of 5 QTL associated with Pro content were identified. Significant *a* ^*e*^ and *a* ^*m*^ were found for all these QTLs, and the additive main effects from maternal plant genome for three QTLs were larger than those from embryo genome. *qPro-LG8-2* with the largest *a* ^*e*^ and *a* ^*m*^ among all QTLs, identified on linkage group 8, could explain a phenotypic variation of 38.3%. Significant *d* ^*e*^, *a* ^*e*^E_1_ and *a* ^*m*^E_1_ showed that the dominance main effect from the embryo genome and environmental factors were not ignored for improving Pro content.

#### Pleiotropism

There was a QTL between markers BNL2496 and NAU855 on chromosome A3, which could simultaneously controlled the performance of Ser, Asp, Ala, Arg, and Pro contents in cottonseeds. The *a* ^*e*^ of the QTL from HS46 could increase Asp, Gly, Ala, and Pro contents but decrease Arg content in cottonseeds. The favorable allele originated from MAR could improve Asp, Ser, Ala, and Pro contents by *a* ^*m*^, while that from MAR could increase Arg content. The environment interaction effects of the QTLs were found in present experiment, implying that the expression of QTL in this locus could be influenced by the environmental conditions. *qSer-5, qGlu-5, qTyr-5, qArg-5*, and *qPro-5* were located between markers RAPD-I10-2000 and NAU3432b on chromosome A5. The genetic effects including additive main effect and dominance main effect from embryo genome were larger than those from maternal genome. The genetic effects from embryo genome from MAR could simultaneously increase Ser, Glu, Tyr, Arg, and Pro contents. *qAsp-18, qSer-18, qGlu-18, qGly-18, qTyr-18, qHis-18, qArg-18*, and *qPro-18* were mapped at the interval between markers TMO409a and NAU1001 on chromosome D18. The *a* ^*e*^ of QTL from HS46 could simultaneously increase Asp, Glu, Tyr, His, Arg, and Pro contents, while it could decrease Ser and Gly contents. *qAsp-22, qAla-22*, and *qArg-22* were identified between markers CIR253 and JESPR65 on chromosome D22. In addition, some QTLs which tend to have pleiotropism were found on other linkage groups as well. This phenomenon could result in some associations among nine amino acids and could improve the traits simultaneously.

## Discussion

Cottonseeds which are produced in large amounts every year could contribute to solving starvation and health problems of an increasing world population (Lambou et al. 1967; Cai et al. 2010). It is for this reason that the improvement of amino acid in cottonseeds has being gaining importance. Amino-acid content in cottonseed is quantitative traits, and their inheritance is very complex (Ji et al. 1988; Huang et al. 2011). However, the complex polygenic quantitative traits can be divided into single Mendelian quantitative trait loci (QTLs) (Paterson et al. 1988; Tanksley 1993). Using QTL mapping can better help understand the genetic architecture of quantitative traits at the molecular level and could further facilitate its improvement through molecular breeding. In this study, a comparative analysis of QTL for nine amino acids was conducted in IF_2_ and BC populations to revealing the key QTLs and their genetic effects. The genetic mechanisms unveiled could provide specific strategy for the improvement of these non-essential amino acids.

Cottonseed is a new generation, different from its maternal plant. The development of cottonseed quality traits is simultaneously controlled by the embryo and the maternal plant genetic systems (Wu et al. 1995; Ye et al. 2003; Liu et al. 2012, 2013). Some experiments have shown that the oil content of cottonseeds was mainly controlled by the maternal plant genetic effect; the protein content was mainly controlled by embryo genetic effect, followed by cytoplasmic effect; while lysine content was mainly controlled by cytoplasmic effects (Wu et al. 1995; Zhu et al. 1997). Zhu and Yu (1995) found that the maternal effect was more important than other genetic effects for protein index in glandless cotton. However, most previous studies especially for QTL were only based on the analysis of dominant and additive effects from the embryo genome (Sigh et al. 1985; Ji and Zhu 1988). Song and Zhang (2007) investigated QTL for four amino acids (Asp, Ser, Gly, and Arg) and a significant QTL *qAsp-A11-1* was detected in a region of 7.6 cm on chromosome A11, explaining 22.12% of phenotypic variation. For Ser, a significant QTL *qSer-A8-1* was identified between NAU1531-170 and NAU537-220, explaining 23.66% of phenotypic variation. For Gly, two significant QTLs *qGly-A11-1* and *qGly-A8-1* were detected with the former explaining 16.97% of phenotypic variation, and the latter, 10.89%. For Arg, a significant QTL was identified in a region of 10.1 cm between two SSR markers NAU1190-205 and NAU797-170 on chromosome A5, explaining 18.21% of phenotypic variation. Only the QTL for Arg was identified on the same chromosome A5 as in our study. These differences may have mainly resulted from the different genetic background of the experimental material used in our studies. In addition, more information including the number, the genetic main effects from embryo and maternal genome, and their environmental interaction effect of QTL for nine amino acids were simultaneously detected.

NIRS (near-infrared reflectance spectroscopy) is a more time-efficient measurement technique. Recently, it has been applied to quantitative and qualitative analyses in various fields. The values of the nine amino-acid contents in the present paper were predicted using NIRS. According to calibration equations for these nine amino acids (Huang et al. 2011), the values are comparable to those obtained by the chemical method, which was validated by verifying a set of 145 samples using a combination of NIRS and the chemical methods.

In this study, the total genetic effects of QTLs for nine amino acids from the maternal plant genome and the embryo genome were divided into different parts. This will help breeders to better understand the genetic mechanism of these traits and adopt a more effective strategy for improving seed quality traits. The results indicated that most QTLs could be simultaneously expressed in different genome-specific tissues, and the expression of these QTLs located on the chromosomes in the embryo and maternal plant was essential for nine non-essential amino acids. Breeders should hence weigh the genetic effects across genetic systems. For example, the embryo main effects including additive and dominance effects from *qAla-22* and *qHis-LG3* were larger than the maternal additive main effect. Thus, embryo genome should be more considered when these two traits are improved. It is applicable to select a single seed of good quality because of the difference among seeds. However, for *qAsp-22* and *qSer-LG5*, the genetic effects from maternal plant genome were larger than those from embryo genome, which showed that the maternal genome was more important for improving these traits in the quality breeding of cottonseed. These two traits can be improved according to the whole performance of the seeds in the maternal plant. Some QTLs (such as *qAla-22* and *qAsp-22*) have either embryo effects or maternal effects, which makes it relatively easier to improve these traits. For some QTLs (such as *qGly-LG5* and *qArg-22*), the additive main effects were more than the dominance main effects, which showed that these traits could be improved more efficiently based on marker-assisted selection (MAS) if tight linkage molecular markers are available. In addition, significant QTL× environment interaction effects from different genetic systems were important and environment factors could not be ignored.

In this present study, some QTLs (such as *qSer-5, qGlu-5, qTyr-5, qArg-5*, and *qPro-5*) for several amino acids were found at the same location. This revealed that some QTLs controlling the performance of different quality traits might be the same or closely located in neighbor positions, which belongs to the phenomena of tight linkage of multigenes or pleiotropy of one gene.

Amino acids are principally divided into two groups. One group cannot be synthesized in the human body and must be obtained from the foods that are eaten, while the other group are important but can be synthesized. Compared to a precious research on eight amino acids which belong to the former group (Liu et al. 2013), some QTLs of some amino acids from two different groups were identified at the same position. For example, *qLeu-5-1, qIle-5, qGlu-5, qTyr-5, qArg-5*, and *qPro-5*, associated with six amino acids, respectively, were all mapped to the interval between markers RAPD-110-2000 and NAU3432b on chromosome 5. Similar results like this could explain the phenotypic relationships among varieties of amino acids. However, for these two types of amino acids, some QTLs were identified on the different chromosomes. This may result in differences among the amino acids. For all the amino acids in these two studies, some QTLs were found to have significant genetic effects from embryo and maternal plant genome, and their environment interaction effects. This results suggested that the genetic effects across genetic systems and environmental factors should be considered when improving these amino acids.

The larger population size provides a greater detection power for the QTLs shared by several crosses (Billotte et al. 2010). Like the *F*_2_ population, the *IF*_2_ population contains abundant information; meanwhile, it could be used to perform repeated trials as an RIL population. These merits are beneficial to marks tightly linked to QTL when *IF*_2_ population is used for QTL analysis. BC population contains the genetic background of a recurrent parent, which makes more minor genes be detected when it is used for QTL identification. Compared with the *IF*_2_ population in the present study, more QTLs with low *R* ^2^ (below 5%) in BC populations were detected in present experiment. For example, for Asp content, the contributions of two QTLs in *IF*_2_ population were much higher than those of all QTLs for this trait in BC population. Thus, BC populations are more suitable for the detection of minor QTL. In the current study, ten QTLs for nine non-essential amino acids were simultaneously detected in these two different kinds of mapping populations, which showed that they could stably express in different mapping populations and these QTLs were more important than others. Due to different softwares for QTL analysis and the genetic differences in these two populations, more individual QTLs could also appear. Among the individual QTLs found in different mapping populations, some QTLs were also important for improving the non-essential amino acids as though they were not common QTLs. Therefore, the method by using two different kinds of populations for QTL mapping could increase the number of QTLs detected, the accuracy of QTL position estimates, and the efficiency of QTL mapping.
